# Cell Attachment to Hydrogel-Electrospun Fiber Mat Composite Materials

**DOI:** 10.3390/jfb3030497

**Published:** 2012-07-27

**Authors:** Ning Han, Jed K. Johnson, Patrick A. Bradley, Kunal S. Parikh, John J. Lannutti, Jessica O. Winter

**Affiliations:** 1 William G. Lowrie Department of Chemical and Biomolecular Engineering, The Ohio State University, Columbus, OH 43210, USA; Email: Ceres-han@hotmail.com (N.H.); kunal.parikh.71@gmail.com (K.S.P.); 2 Department of Materials and Science Engineering, The Ohio State University, Columbus, OH 43210, USA; Email: jed.johnson@nanofibersolutions.com (J.K.J.); lannuttj@matsceng.ohio-state.edu (J.J.L.); 3 Department of Biomedical Engineering, The Ohio State University, Columbus, OH 43210, USA; Email: bradley.317@osu.edu

**Keywords:** hydrogels, electrospun fibers, cell attachment, nanotopography, composite materials

## Abstract

Hydrogels, electrospun fiber mats (EFMs), and their composites have been extensively studied for tissue engineering because of their physical and chemical similarity to native biological systems. However, while chemically similar, hydrogels and electrospun fiber mats display very different topographical features. Here, we examine the influence of surface topography and composition of hydrogels, EFMs, and hydrogel-EFM composites on cell behavior. Materials studied were composed of synthetic poly(ethylene glycol) (PEG) and poly(ethylene glycol)-poly(ε-caprolactone) (PEGPCL) hydrogels and electrospun poly(caprolactone) (PCL) and core/shell PCL/PEGPCL constituent materials. The number of adherent cells and cell circularity were most strongly influenced by the fibrous nature of materials (e.g., topography), whereas cell spreading was more strongly influenced by material composition (e.g., chemistry). These results suggest that cell attachment and proliferation to hydrogel-EFM composites can be tuned by varying these properties to provide important insights for the future design of such composite materials.

## 1. Introduction

Because of their similarity to biological structures, both hydrogels [1] and electrospun fiber mats (EFMs) [2] have been widely used for tissue engineering. Hydrogels, which are formed from crosslinked hydrophilic polymers, display high water content, low mechanical modulus, and biocompatibility, making them attractive as soft tissue engineering constructs [3]. Similarly, EFMs, formed by applying a voltage to charged polymer solutions [4,5], have attracted interest as tissue engineering scaffolds because of their fibrous nature, which mimics components of the extracellular matrix (ECM) (e.g., nm-µm fiber diameters) [6,7], and their high surface area-to-volume ratio, which increases cell contact area [7]. These materials can be tuned to structurally and mechanically resemble native ECM by altering either EFM or hydrogel properties; both can support cell attachment and proliferation [6,8]. Recently, several researchers have combined the benefits of these two materials, which may be either synthetic or naturally-derived, to yield hydrogel-electrospun fiber composites for applications ranging from neural tissue engineering to arterial bypass grafts [9,10,11,12,13,14]. Of particular interest in this study is their application as neural prosthesis coatings to enhance device biocompatibility [9,15]. However, the relationship between cell attachment and specific chemical and topographical features in these composites has yet to be explored. 

Initial cell attachment to a biomaterial can determine cell fate, including viability, proliferation, and function [16], and is thus an important component of the overall cell response to biomaterials. If normally adherent cells do not attach, they will likely undergo apoptosis and die. Cells that attach, but do not spread, may also incur a similar fate. When adherent cells in tissues contact a biomedical implant, fibrotic scar tissue can form. Resistance to cell attachment can be an important modulator of this behavior [17,18,19]. The interaction of adherent cells with artificial substrates can be divided into four separate steps: (1) cell attachment; (2) spreading; (3) actin cytoskeleton organization; (4) focal contact formation [20]. *In vivo*, cell attachment to the ECM occurs in response to chemical and topographical cues [21]; however, it can be difficult to distinguish between these effects *in vitro*.

The hydrogel-EFM system offers a unique model to explore the effects of surface chemistry and topography in cell attachment. Hydrogels consist of entangled or cross-linked polymer chains that have an amorphous to semi-fibrous character, whereas EFMs display distinct nano- to micro-scale topography that can be easily tuned [22]. Thus, EFMs and hydrogels can be manufactured from polymers of the same composition, but with dramatically different topographical features. The goal of this study was to exploit the topographical differences between same composition hydrogels, EFMs and composites to examine the influence of materials chemistry *versus* topography on cell attachment.

Specifically, we examined cell attachment to synthetic hydrogel-EFM composite materials composed of poly(ethylene glycol) (PEG) and poly(caprolactone) (PCL), two commonly used biomaterials. PEG generally resists cell attachment as a result of its high favorability for binding water [23] and is often referred to as a “stealth material” because of its resistance to protein adsorption [3]. PCL is a widely-used biodegradable polyester. PEG is highly hydrophilic, whereas PCL is much more hydrophobic and is therefore not conducive to cell growth [24]. However, cell attachment can be enhanced by the introduction of nanoscale topographical features, such as those presented by the EFMs [25,26]. Neither polymer has biological activity. Cell attachment and proliferation were compared for PEG and PCL-based hydrogel, EFM, and composite materials, and the influence of topography and chemical composition on cell behavior examined. The results of this work can be used to better inform future design of hydrogel-EFM composites and suggest a potential method to increase adhesion to hydrogels that do not normally support cell adhesion through the introduction of nanotopography.

## 2. Results and Discussion

We compared cell attachment and proliferation on materials from three categories (Table 1): hydrogels, EFMs, and their composites. For hydrogel (G) materials, we investigated PEG and PEGPCL diacrylates as PCL itself is too hydrophobic to effectively form a hydrogel independently. As EFM materials, we investigated PEGPCL and PCL in two configurations: monolithic fibers of PCL and core/shell fibers with PEGPCL as the surface “shell” and PCL as the interior fiber “core” (PCL/PEGPCL core/shell). Composites were formed using PEGPCL Gs, as PEG Gs did not support cell attachment, and PCL and PCL/PEGPCL EFMs. Thus, PEGPCL Gs, PCL/PEGPCL EFMs, and PEGPCL G-PCL/PEGPCL EFM composites all possess the same outward PEGPCL surface chemistry while displaying different topographical features (gel, fibers, and composite). Conversely, PEG Gs, PCL EFMs, and PEGPCL G-PCL EFM composites offer similar topography but differing compositions to materials in the same category. 

**Table 1:** Materials Investigated.

Material ^1^	Topography	Surface Composition
PEG G	Gel	PEG
PEGPCL G	Gel	PEGPCL
PCL EFM	Fibers	PCL
PCL/PEGPCL ^2^ EFM	Fibers	PEGPCL
PEGPCL G-PCL EFM	Composite	PCL
PEGPCL G-PCL/PEGPCL^2^ EFM	Composite	PEGPCL

Notes: ^1 ^G = Hydrogel, EFM = Electrospun Fiber Mat; ^2 ^EFM core material/shell material.

Hydrogel-EFM composite materials are being investigated as coatings for neural prosthetic devices to enhance biocompatibility between implanted electrodes and host tissue [9,15]. Thus, consistent with this goal, cell attachment analyses were performed using two model cell lines: SK-N-SH human neuroblastoma cells and rat cortical primary cells. SK-N-SH cells were chosen because they are known to exhibit two distinct morphologies depending on the adherent surface [27] and are thus potentially responsive to topographical changes, and rat cortical primary cells were selected to provide a model as close as possible to that found *in vivo*.

### 2.1. Characterization of Electrospun Fiber Mats

**Figure 1:**
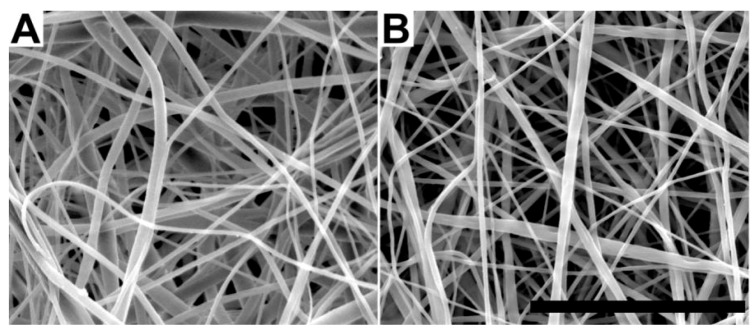
Scanning electron micrographs of (**a**) poly(caprolactone) (PCL) and (**b**) PCL/poly(ethylene glycol)-poly(ε-caprolactone) (PEGPCL) core/shell electrospun fiber mats (EFMs). Scale bar: 50 µm.

The primary purpose of this study was to examine the role of surface chemistry *versus* topography in directing cell attachment and proliferation in hydrogel-EFM composites for neural tissue engineering. It is therefore critical that the fibers employed display similar topographical properties after surface chemistry modification. Thus, in addition to water contact angle, which establishes hydrophobicity of the fiber surface, average fiber diameter, mean pore size, and porosity (Table 2, Figure 1) were determined for both PCL and PCL/PEGPCL core/shell randomly aligned EFM scaffolds. The water contact angles of PCL and PCL/PEGPCL EFMs were 135.2 ± 5.4° and ~0°, respectively, establishing these materials as example hydrophobic (PCL) and hydrophilic (core/shell PCL/PEGPCL) EFM substrates. Morphological characterization via scanning electron microscopy (SEM) (Figure 1) showed no significant difference in average fiber width and mean pore size between PCL and PCL/PEGPCL EFMs (*p* > 0.05). Similarly, no significant difference of porosity (*p* > 0.05), as measured via ethanol displacement, was found between PCL and PCL/PEGPCL EFMs. The “fractional contact area”, Φ, which is the fraction of fiber surface contacting other fibers, was used to establish the “available surface fraction”, (1 − Φ), open to cell adhesion. The available surface fraction was calculated from ε based on the theory of Eichhorn and Sampson [28] and assuming a mean coverage of ~10 to ∞ based on SEM data. These values suggest that these materials are suitable as tissue engineering constructs as >70% of the surface is available. Thus, the EFMs employed exhibited nearly identical morphologies, but altered surface properties (*i.e.*, composition and hydrophobicity). 

**Table 2:** Comparison of variables of EFMs.

EFM	Water contact angle (°)	Average fiber width ω (µm)	Mean pore size (µm)	Porosity ε (%)	Available surface fraction (1 − Φ)
PCL	135.2 ± 5.4	1.726 ± 0.102	1.517 ± 0.203	82 ± 2	~0.79
PCL/PEGPCL	~0	1.512 ± 0.141	1.557 ± 0.229	83 ± 2	~0.73

Note: Data are expressed as mean ± standard deviation.

### 2.2. SK-N-SH Neuroblastoma Cell Attachment and Proliferation

**Figure 2:**
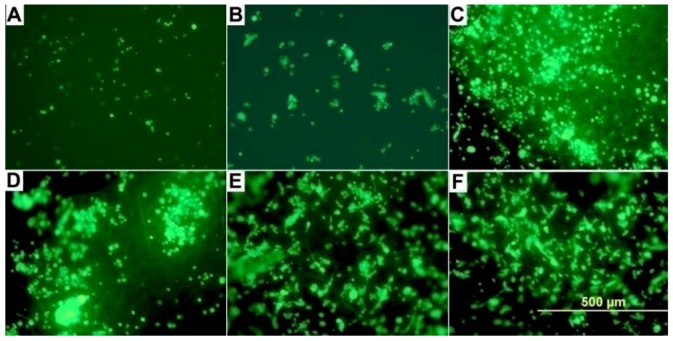
Reflected DIC fluorescent micrographs of SK-N-SH cells on (**a**) PEG G; (**b**) PEGPCL hydrogel (G); (**c**) PEGPCL G-PCL EFM composite; (**d**) PEGPCL G-PCL/PEGPCL EFM composite; (**e**) PCL EFM; (**f**) PCL/PEGPCL EFM materials. Scale bar: 500 µm.

SK-N-SH neuroblastoma cell attachment was examined on two materials with differing topographical features: Gs and EFMs, and two materials with differing hydrophobicity in each group: PEG < PEGPCL for Gs and PEGPCL < PCL for EFMs. We also investigated composites of these two materials that were formed by *in situ* photopolymerization to secure EFMs to the outer hydrogel surface, thus presenting EFMs as the primary surface available for cell attachment (see Table 1 for all materials employed). Cell attachment and proliferation were qualitatively evaluated using fluorescently-labeled samples after 1 day of incubation, which is sufficient to permit attachment, but unlikely to result in substantial proliferation. Qualitatively, it is clear that cells preferred composite (Figure 2C, D) and EFM (Figure 2E, F) surfaces to G surfaces (Figure 2A, B), despite the fact that the PEGPCL G (Figure 2B), PEGPCL G-PCL/PEGPCL EFM composites (Figure 2D), and PCL/PEGPCL EFMs (Figure 2F) present identical PEGPCL surface compositions. 

**Figure 3:**
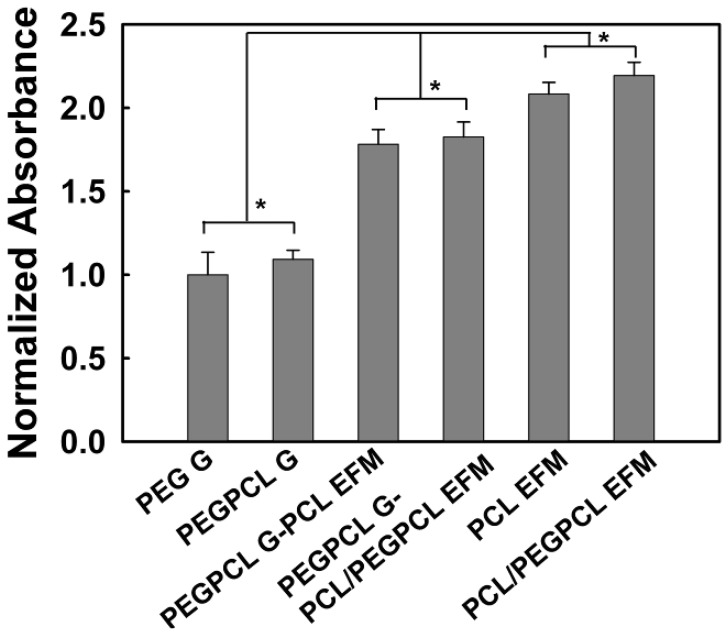
SK-N-SH cell attachment to and proliferation on hydrogel (G), electrospun fiber mat (EFM), and composite materials after 24 hours. The asterisk indicates significant difference (*p* > 0.05). Data normalized to that of PEG G.

To further quantify these results, SK-N-SH cell proliferation was evaluated using the MTT assay (Figure 3). Interestingly, there were no significant differences in cell number as measured via normalized absorbance between Gs, EFMs, and composites of differing surface chemistry (*i.e.*, more hydrophilic *vs.* more hydrophobic). However, significant differences were observed between any two of these materials (*p* < 0.05). Thus, the number of adherent cells was highest for materials that displayed more fibrous character (e.g., EFM > composites > hydrogels), regardless of the hydrophilicity of the component materials (e.g., PEG > PEGPCL > PCL); and the incorporation of EFMs increased cell attachment to hydrogels composed of the same materials. These results most likely occur because of differences in topography; however, the increased adhesive surface area of EFMs could also contribute. EFMs display pore sizes of ~1–2 µm (Table 2), in contrast, hydrogels have pore sizes on the order of a few nanometers [29], and would thus appear as more two-dimensional surfaces than the EFMs. 

**Figure 4:**
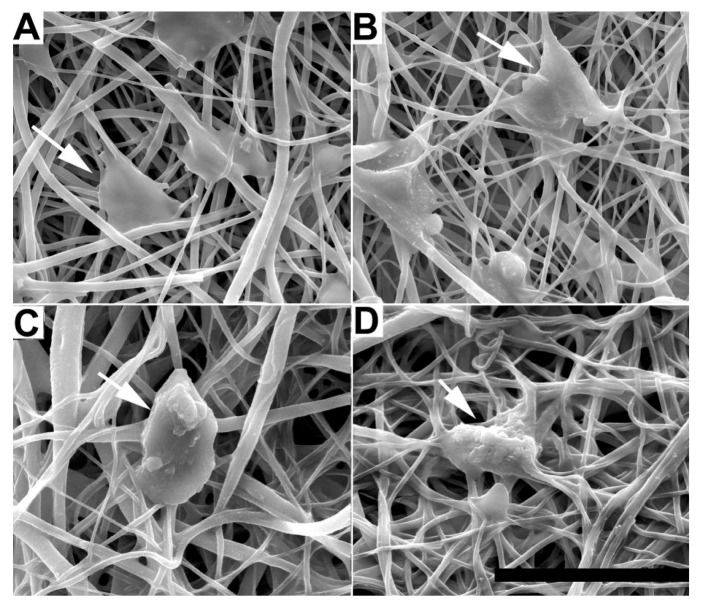
SEM images of SK-N-SH cells on (**A**) PCL; (**B**) PCL/PEGPCL EFMs; (**C**) PEGPCL G-PCL EFM; (**D**) PEGPCL G-PCL/PEGPCL EFM composites. Scale bar: 50 µm. Arrows indicate representative cells.

**Figure 5:**
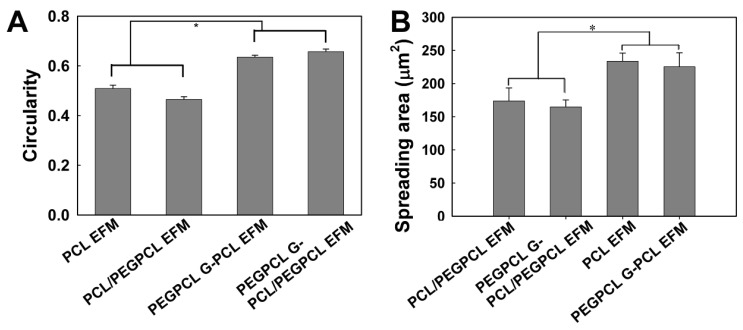
(**A**) Circularity; (**B**) spreading of SK-N-SH cells cultured on EFM and composite materials. One asterisk indicates significant difference between samples.

The morphology of adherent cells was also examined on EFM and composite surfaces using qualitative SEM imaging (Figure 4) and quantification of cell spreading and circularity using ImageJ image analysis software (Figure 5A, B). G surfaces were not examined because of the low number of adherent cells (e.g., Figure 2). In all SEM images, cells spread on EFM and composite surfaces, aligning to and interacting with multiple fibers. However, cells on composite materials (Figure 4C, D) appeared more rounded than those on EFM-only surfaces (Figure 4A, B), despite the similarities in surface composition between materials that displayed PCL (Figure 4A, C) and PCL/PEGPCL EFMs (Figure 4B, D), respectively.

Cell circularity and spreading values further describe these morphological observations. Circularity is measured on a scale of 0 to 1, with 1 representing a completely circular cell and 0 representing a completely polarized cell. Cell spreading is measured by quantifying the surface area coverage of cells and is measured in µm^2^. Consistent with SEM observations (Figure 4), the circularity of SK-N-SH cells on EFM-only scaffolds was significantly lower than that of cells on composite scaffolds (*p* < 0.05) (Figure 5A), despite similarities in material surface composition (e.g., PCL EFM *vs.* PEGPCL G-PCL EFM composite and PCL/PEGPCL EFM *vs.* PEGPCL G-PCL/PEGPCL EFM composite). This suggests a topographical influence; however, the role of mechanical properties cannot be excluded. PCL EFMs have a modulus of ~7 MPa [30], whereas PEGPCL hydrogel moduli are on the order of ~0.1–1 MPa [29]. Mechanical properties, including increased stiffness, have been shown to influence cell spreading, proliferation, and migration [31,32].

In contrast to other observed parameters, cell spreading (Figure 5B) was primarily influenced by composition. The more hydrophobic PCL EFM-based materials (PCL EFM and PEGPCL G-PCL EFM composite) displayed significantly higher average spreading values than PCL/PEGPCL EFM-based samples (PCL/PEGPCL EFM and PEGPCL G-PCL/PEGPCL EFM composite) (*p* < 0.05), whereas there was no significant difference in cell spreading within groups potentially presenting different topographies but similar surface characteristics (PCL EFM *vs.* PEGPCL G-PCL EFM; or PCL/PEGPCL EFM *vs.* PEGPCL G-PCL/PEGPCL EFM composite) (*p* > 0.05).

### 2.3. Rat Cortical Cell Attachment to Composite Materials

**Figure 6:**
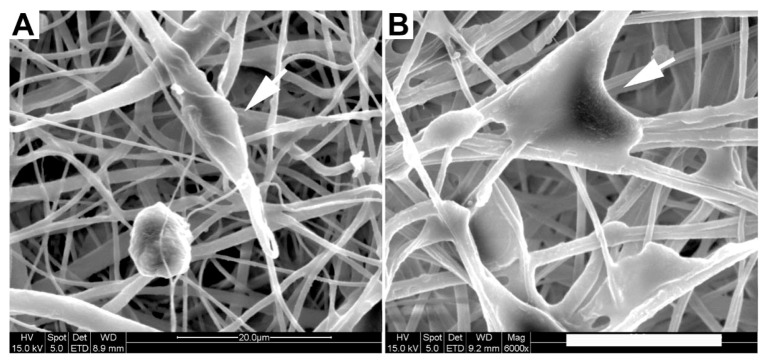
SEM images of rat cortical cells on (**a**) PEGPCL G-PCL EFM; (**b**) PEGPCL G-PCL/PEGPCL EFM composite materials. Arrows indicate representative cells. Scale bar: 20 µm.

**Figure 7:**
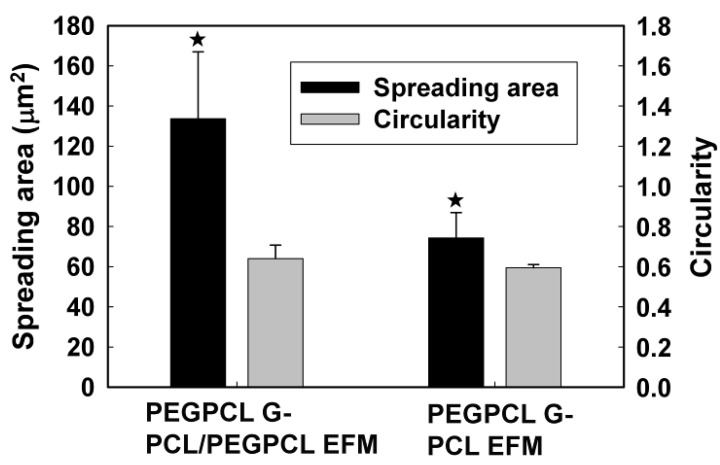
Spreading area and circularity of primary rat cortical cells on PEGPCL G-PCL/PEGPCL EFM and PEGPCL G-PCL EFM composite materials. One asterisk indicates significant difference between pair samples.

To further evaluate composite materials (PEGPCL G-PCL EFM and PEGPCL G-PCL/PEGPCL EFM composites) in a more physiologically relevant system, we also explored the behavior of adherent primary rat cortical neurons. SEM images (Figure 6) show that cells on the more hydrophilic PEGPCL G-PCL/PEGPCL EFM composite samples exhibited greater spreading, indicating better interaction between cortical cells and this scaffold. This was further validated by quantification of cell spreading. Cells on more hydrophilic PEGPCL G-PCL/PEGPCL EFM composite surfaces showed significantly larger spread areas than those cultured on PEGPCL G-PCL EFM composite materials (*p* < 0.05) (Figure 7). Interestingly, although surface chemistry was still a driving factor in cell spreading, the trend observed with cortical cells was opposite to that of SK-N-SH cells. More hydrophilic surfaces (*i.e.*, PEGPCL G-PCL/PEGPCL EFM composites) favored cell spreading of rat cortical cells (Figure 7), whereas SK-N-SH cells spread more on hydrophobic surfaces (Figure 5B). No significant difference was observed in cell circularity between these two samples (*p* > 0.05) (Figure 7) consistent with the results of SK-N-SH culture (Figure 5A).

## 3. Experimental Section

### 3.1. Electrospun Fiber Mat Fabrication

All chemicals were purchased from Sigma-Aldrich, unless stated otherwise.

#### 3.1.1. PCL Mat Fabrication

A 5 wt% solution of poly(ε-caprolactone) (PCL) in 1,1,1,3,3,3-Hexafluoro-2-propanol (HFIP) was prepared by continuous stirring at room temperature overnight to dissolve the PCL. This solution was then placed in a 60 cc syringe with a 20 gauge blunt tip needle and electrospun using a high voltage direct current power supply (Glassman High Voltage, Inc., High Bridge, NJ, USA) set to +26 kV, a 20 cm tip-to-substrate distance and a 15 mL/h flow rate. A 3 × 3'' (7.6 × 7.6 cm) sheet ~0.2 mm in thickness was deposited onto aluminum foil. PCL sheets were then placed under vacuum overnight to ensure removal of residual HFIP.

#### 3.1.2. PCL/PEGPCL Core/Shell Mat Fabrication

A core solution of 18 wt% PCL in acetone was prepared by continuous stirring at room temperature overnight to dissolve the PCL. Meanwhile, a shell solution of 50 wt% PEGPCL copolymer [33] in acetone was prepared by continuous stirring at room temperature overnight to dissolve the PEGPCL copolymer. Core/shell electrospinning was carried out as described previously [30] except the core flow rate was 10 mL/h, whereas the shell flow rate was 1 mL/hr. Shell continuity was validated as described previously using X-ray photoelectron spectroscopy (XPS) [30].

PCL and PCL/PEGPCL core/shell EFMs were cut into circular discs with a diameter (D) of 6 or 8 mm and a thickness of approximately 200–300 µm using derma punches (Acuderm). For cell culture studies, all matrices were soaked in 70% ethanol for 2 hours, dried, and sterilized under UV light in a sterile hood overnight. To evaluate the effect of EFMs on cell attachment and viability, EFMs were secured to the bottom of tissue culture plates using carbon double-side adhesive tape (Ted Pella, Inc., Redding, CA, USA) and immersed in cell culture media for 24 hours before cell seeding. The carbon double-sided adhesive tape was tested to ensure no negative effect on cell proliferation (data not shown).

### 3.2. Electrospun Fiber Mat Characterization

To evaluate EFM hydrophobicity and surface properties, EFM water contact angles were measured at room temperature with a contact angle goniometer (Ramé-hart Instrument co., Model 200, Succasunna, NJ, USA). At least 5 measurements were taken per condition. The morphologies (*i.e.*, fiber width and pore size) of PCL and PCL/PEGPCL core/shell electrospun fiber mats were characterized by scanning electron microscopy (SEM, Quanta 200, FEI). All samples were secured to aluminum mounts by double-sided carbon tape and then sputter coated with gold before viewing. SEM images at 800×, 1500× and 3000× magnifications (N = 3) were analyzed using Image J (NIH) to measure the fiber width (ω) and pore size. For fiber width measurements, at least 75 fibers per sample and 3 samples per condition were examined. For pore size measurements, at least 50 pores per sample and 3 samples per condition were analyzed to obtain the average value of the major axis for these noncircular pores [34]. 

Network porosity (ε), was estimated using a liquid displacement method reported by Guan *et al.* [35]. Ethanol was selected as the displacement liquid because of its ease of penetration into open pores without inducing swelling or shrinkage. EFM samples were immersed in a known volume of ethanol (V_1_) for 5 min, and then pressed to force air from open pores. The total volume of ethanol and immersed samples was recorded as V_2_. After the removal of ethanol-impregnated samples, the volume of the residual ethanol was recorded as V_3_. EFM porosity was calculated using the following Equation:

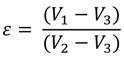
(1)

### 3.3. Synthesis of Diacryl-PEGPCL Copolymer

Diacryl-PEGPCL copolymer was synthesized as described previously [33], using a modified method derived from Hubbell *et al.* [36]. Briefly, in the presence of stannous octoate (Tin II-ethylhexanoate), new chemical bonds between ε-caprolactone and the hydroxyl ends of toluene-azeotropic distilled PEG (molecular weight M_w_ = 950–1,050) were formed through ring-opening polymerization at 130 °C under an argon atmosphere. The molar ratio of ε-caprolactone to PEG was 2 to 1. Then, after being precipitated in ice-cold hexane, filtered through a Buchner funnel, and dried in a vacuum oven for 1 hour at 40 °C, purified PEGPCL intermediate was acrylated using acryloyl chloride (ACCl). Under argon atmosphere, the mixture of ACCl and dichloromethane (DCM) was slowly added (0.2 mL/min) to the mixture of PEGPCL intermediate, triethylamine (TEA), and DCM in dry ice-acetone bath ~−30 °C. After the addition of ACCl, the reaction was completed through ~60 hours continuous stirring at room temperature. Byproduct TEA-HCl was removed by repeated solvent evaporation and filtration. Diacryl-PEGPCL product was precipitated in ice-cold diethyl ether, filtered through a Buchner funnel and dried in a vacuum oven in the presence of P_2_O_5_ overnight. Purified PEGPCL intermediate and diacryl-PEGPCL polymer could be stored in a −20 °C freezer for up to a month. Proton nuclear magnetic resonance (H^1^ NMR, Bruker DPX400) and Fourier transform infrared resonance (FT-IR, Thermo Scientific) were applied to characterize copolymer samples. The yields of PEGPCL intermediate and diacryl-PEGPCL were 91.3% and 85.2% respectively, consistent with previously reported results [33].

### 3.4. Formation of Hydrogels and Hydrogel-Electrospun Fiber Mat Composite Materials

Diacryl- PEG or PEGPCL polymer was dissolved in Dulbecco’s phosphate buffered saline (D-PBS) at 22 wt %, and then mixed with initiators (0.1 wt % Irgacure 2959 and 0.5 wt % 1-vinyl-2-pyrrolidone). Precursor solution was sterilized via filtration (0.22 µm filter with 0.8 µm pre-filter, Millipore) before UV photopolymerization. 

#### 3.4.1. PEG and PEGPCL Hydrogels

Fifty microliters of PEG or PEGPCL precursor solution was pipetted into a single well of a 96-well tissue culture plate (BD Biosciences), followed by 7 min of UV exposure (Blak-Ray, B-100-AP, UVP). To remove potential residual chemicals, materials were then rinsed with 150 µL phosphate buffered saline (PBS) solution per well and incubated (37 °C, 5% CO_2_) for one hour. Following PBS solution aspiration, hydrogels were immersed in cell culture media for 24 hours prior to cell seeding. 

#### 3.4.2. Composite Materials

Composite materials were prepared as described previously [37]. For SK-N-SH cells, 50 µL PEGPCL precursor solution was added into a single well of a 96-well tissue culture plate, and then exposed to UV light for 6 min. Before the precursor solution fully gelled, an EFM (D = 6 mm) was placed in the same well, followed by 1.5 min of UV exposure. For rat cortical neuron culture, an EFM (D = 8 mm) was placed in an 8 mm well of an 8-well SecureSeal^TM^ hybridization chamber (Grace, Bio-labs, SA8R-2.5), followed by the addition of 50 µL of PEGPCL precursor solution. After 6.5 min of UV illumination, a second EFM was placed in the same well. Composite construction was then completed with 1 min of additional UV illumination. Composite materials were secured to the bottom of a well in a 24-well tissue culture plate (2 composites per well) using a sterile bio-glue (Dow Corning, Silastic Medical Adhesive Silicone, Type A). PBS solution and cell culture media immersion were used to remove residual chemicals from composite materials in both SK-N-SH and cortical cell culture experiments as described above. 

### 3.5. SK-N-SH Cell Culture

SK-N-SH cells were purchased from American Type Culture Collection (ATCC, CRL-1721, Manassas, VA, USA). Cells were cultured in Dulbecco’s modified Eagle’s medium (DMEM) containing 10% fetal bovine serum (FBS, Gibco) and 1% penicillin-streptomycin (Gibco) as per the manufacturer’s instructions. PEG and PEGPCL Gs, PEGPCL G-PCL EFM and PEGPCL G-PCL/PEGPCL EFM composites, and PCL and PCL/PEGPCL EFMs were deposited in a 96-well tissue culture plate as described previously (N = 6 for each material). SK-N-SH cells were seeded at a density of 5 × 10^4^ cells/cm^2^ into each well and cultured overnight in an incubator (37 °C, 5% CO_2_). 

### 3.6. SK-N-SH Cell Fluorescent Imaging and the MTT Proliferation Assay

SK-N-SH cell attachment and viability on these materials was evaluated using fluorescent cell tracking (N = 3), the MTT cell proliferation assay (N = 3), and SEM imaging (N = 3, as described in Section 3.8). For fluorescent cell tracking, SK-N-SH cells were pre-stained using the Celltracker^TM^ fluorescent imaging probe (Green CMFDA, Invitrogen). After 1 day, fluorescently-labeled SK-N-SH cells were imaged using a reflected differential interference contrast (DIC) microscope (Olympus BX41). Five images per magnification (10×, 20×) were taken at random spots for each sample (N = 3). The MTT cell proliferation assay was conducted following standard protocols (Sigma-Aldrich). Sample absorbance was observed using a Versamax UV-visible micro-plate reader in triplicate.

### 3.7. Rat Cortical Neuron Culture

Rat cortical neurons (from E18 rat cortex) were purchased from BrainBits LLC. PEGPCL G-PCL EFM and PEGPCL G-PCL/PEGPCL EFM composites were examined in cortical culture. Hydrogel-EFM composite materials (N = 3 for each sample) were sterilized and secured to the bottom of wells in sterile 24-well tissue culture plates as described previously. Rat cortical neurons were seeded at 5 × 10^5^ cells/cm^2^, cultured in neurobasal growth medium with 2% B-27, 0.5mM Glutamax added (all Invitrogen), and incubated at 37 °C, 5% CO_2_ for 4 days before morphological evaluation. Cortical cultures were examined 4 days after seeding, as opposed to the 24 hour period used for SK-N-SH experiments, to permit maturation of the embryonic cells to the neural phenotype [38,39].

### 3.8. Cell Fixation and Scaffold SEM Imaging

SK-N-SH cells were fixed after 1 day of incubation. After rinsing residual proteins with a PBS wash, cells were fixed in PBS containing 4% paraformaldehyde and 4% sucrose for 1 hour at room temperature. Samples were then rinsed three times with PBS containing 4% sucrose, and dehydrated in a graded ethanol series (50, 70, 80, 95 and 100% for 5, 5, 10, 10 and 10 × 2 min, respectively) and ethanol:hexamethyldisilazane (HMDS, Sigma-Aldrich) series (75, 50, 25 and 0% Ethanol remainder HMDS for 15, 15, 15 and 15 × 2 plus overnight, respectively). After completely dehydrated, scaffolds were mounted on specimen holders, sputter-coated with gold, and observed using SEM (Quanta 200, FEI). Images were collected at 800×, 1,500× and 3,000× (N = 6) magnifications and analyzed using Image J software (NIH, Bethesda, MD, USA). Cell area and cell circularity were measured by selecting individual cells and obtained by setting the measurements to report area and shape descriptors. Similarly, after a 4 days incubation period, the same fixation, dehydration, and SEM imaging procedures were applied to characterize rat cortical neuron seeded scaffolds. 

### 3.9. Statistical Analysis

Statistical analyses were performed using one way analysis of variance (ANOVA) with Tukey HSD testing (JMP 8.0). *p* values of <0.05 were considered to be significant. 

## 4. Conclusions

The primary focus of this study was to compare the effects of chemical and topographical cues on cell behavior using hydrogel-electrospun fiber mat composites. To investigate these influences, hydrogels, EFMs, and hydrogel-EFM composites composed of PEG, PCL, and PEGPCL polymers were examined. The structural characteristics of hydrogels were not directly evaluated by electron microscopy as their high water content makes it difficult to perform these analyses under the vacuum conditions required. However, Figure 4 and Figure 7 provide some indication of the contribution of hydrogels to surface morphology. In these figures, there is no clear difference in topography displayed with the addition of hydrogels, suggesting that EFMs contribute far more strongly to surface nanotopography than hydrogels. Hydrogels of this type (e.g., PEG-hydroxy acids) generally form dense, tightly cross-linked, porous structures with pore sizes on the order of a few nm [29], and thus effectively appear as a roughened surface to a ~10 µm cell. EFMs employed used a core/shell structure, which permitted surface hydrophobicity and chemistry to be altered while keeping fiber structure (e.g., diameter, porosity, and modulus) relatively constant (Figure 1, Table 2). The much larger pore sizes and fiber diameters of EFMs (~1 µm, Table 2, Figure 1) are within the same order of magnitude as the cell diameter and provide topographical features with which cells can interact. These materials thus provide the same surface chemistry (e.g., PEG, PCL, or PEGPCL composition), but varying topographical features and also adhesive area.

Cell responses to these materials suggested that initial cell attachment and circularity were more strongly influenced by changes in topography than surface chemistry (Figure 3 and Figure 5). There are several possible explanations for this observation, including the likely increased surface area presented for cell attachment, the influence of nanoscale features that may increase protein adsorption [40,41], and the potential role of mechanics [42]. Mechanical modulus can influence cell behavior, including cell adhesion, proliferation, and ultimately cell fate [31,32]. The mechanical modulus of the EFMs employed is ~7 MPa [30], whereas that of PEG-based hydrogels is on the order of 0.01–1 MPa [29]. It is therefore possible that cells preferred EFMs to hydrogels because of their increased modulus; however, the clear influence of EFMs on cell circularity suggests that topography does influence at least some cell attachment and spreading behaviors (Figure 5A). This is consistent with previous work, which has shown that cell alignment is extremely sensitive to nanoscale patterns [43], including those presented by electrospun nanofibers [44]. In contrast, cell spreading was found to depend more strongly on chemical composition than topography, although opposite effects were observed for SK-N-SH *versus* rat cortical cells (Figure 5B and Figure 7). One possible explanation for these divergent behaviors is the role of protein adsorption in cell adhesion and spreading. SK-N-SH cells were cultured in the presence of serum, whereas rat cortical neurons were cultured in serum-free medium (with Glutamax supplement). Because different proteins were most likely present in both solutions, biomaterial surfaces probably presented different adsorptive protein profiles to cells. However, the preference of SK-N-SH cells for more hydrophobic PCL substrates is also in contrast with previous observations [45], and suggests that hydrophobicity alone was not the driving feature in SK-N-SH cell preference for these surfaces. 

These studies demonstrate that both chemical composition and topography can influence cell attachment and proliferation on hydrogel-EFM composites and provide important insight into the design of these tissue engineering constructs. Further, this work suggests a possible method to increase cell attachment to surfaces that do not typically support adhesion by the addition of nanotopographical features. These results confirm that topography, even if randomly oriented, can influence cell behavior; aligned substrates could produce even stronger topographical responses. Also, the influence of EFM spatial properties (e.g., pore size, fiber density and diameter) was not evaluated and would likely play a role. To further increase chemical sensitivity, advanced EFM fabrication techniques, such as surface modification with adhesion molecules or co-spinning with natural ECM polymers, could be applied. Hydrogel-EFM composites thus present an exciting new material for tissue engineering with the potential for tunable cell adhesion through both topographical and chemical cues.
